# Hôpital de Saint Louis

**Published:** 1833-01

**Authors:** 


### HOPITAL DE SAINT LOUIS.

### *Injury of the First Pair of Nerves, followed by Loss of Smell.*

Among several cases of gunshot wounds admitted into the Hôpital de Saint-Louis, reports of which have been made by M. Voisin (*interne de l'hôpital,*) the following is physiologically interesting:

In this case, the ball had passed through both orbits in the plane of the root of the nose, destroying the two eyeballs and the olfactory nerves. The sense of smell is entirely lost. Vinegar, strong ammonia, and so forth, have been employed without any effect to produce their ordinary results. This case is reported for the benefit of those modern physiologists who would dispossess the olfactory nerves (the first pair) of their ancient privileges, for the special aggrandisement of the Trigemini.—*Ibid.*

### COLLECTANEA.

